# Issues in the incorporation of economic perspectives and evidence into Cochrane reviews

**DOI:** 10.1186/2046-4053-2-83

**Published:** 2013-09-20

**Authors:** Ian Shemilt, David McDaid, Kevin Marsh, Catherine Henderson, Evelina Bertranou, Jacqueline Mallander, Mike Drummond, Miranda Mugford, Luke Vale

**Affiliations:** 1 Behaviour and Health Research Unit, University of Cambridge, Robinson Way Cambridge CB2 0SR, UK; 2 Personal Social Services Research Unit, London School of Economics, Houghton Street, London WC2A 2AE, UK; 3 United BioSource Corporation, 26-28 Hammersmith Grove, London W6 7HA, UK; 4 Matrix Knowledge, 152-160 City Road, London EC12 2NP, UK; 5 Centre for Health Economics, University of York, Heslington York YO10 5DD, UK; 6 Health Economics Group, University of East Anglia, Norwich Research Park, Norwich NR4 7TJ, UK; 7 Institute of Health & Society, Newcastle University, Richardson Road, Newcastle, Upon Tyne NE2 4AX, UK

**Keywords:** Cost-utility analysis, Cost-effectiveness analysis, Systematic review, Meta-analysis, Cochrane collaboration

## Abstract

**Background:**

Methods for systematic reviews of the effects of health interventions have focused mainly on addressing the question of 'What works?’ or 'Is this intervention effective in achieving one or more specific outcomes?’ Addressing the question 'Is it worth it given the resources available?’ has received less attention. This latter question can be addressed by applying an economic lens to the systematic review process.

This paper reflects on the value and desire for the consideration by end users for coverage of an economic perspective in a Cochrane review and outlines two potential approaches and future directions.

**Methods:**

Two frameworks to guide review authors who are seeking to include an economic perspective are outlined. The first involves conducting a full systematic review of economic evaluations that is integrated into a review of intervention effects. The second involves developing a brief economic commentary. The two approaches share a set of common stages but allow the tailoring of the economic component of the Cochrane review to the skills and resources available to the review team.

**Results:**

The number of studies using the methods outlined in the paper is limited, and further examples are needed both to explore the value of these approaches and to further develop them. The rate of progress will hinge on the organisational leadership, capacity and resources available to the CCEMG, author teams and other Cochrane entities. Particular methodological challenges to overcome relate to understanding the key economic trade-offs and casual relationships for a given decision problem and informing the development of evaluations designed to support local decision-makers.

**Conclusions:**

Methods for incorporating economic perspectives and evidence into Cochrane intervention reviews are established. Their role is not to provide a precise estimate of 'cost-effectiveness’ but rather to help end-users of Cochrane reviews to determine the implications of the economic components of reviews for their own specific decisions.

## Background

Methods for systematic reviews of health interventions have focused mainly on addressing the question of 'What works?’ or 'Is this intervention effective in achieving one or more specific outcomes?’ Systematic reviews have addressed the efficiency of those interventions less often. Yet such questions as 'Is it worth it?’, 'At what cost is the outcome achieved?’, and 'What will be the economic impact of this intervention?’ are crucial if health systems are to use the resource they have available to their best advantage. In times of financial austerity, these questions take on particular importance. In his seminal work *Effectiveness and Efficiency: Random Reflections on Health Services* (authors’ emphasis), Archie Cochrane stressed, as shown in the quotation below, the vital role of economic evidence in health decision making [1]. As The Cochrane Collaboration celebrates its 20th Anniversary, we consider the extent to which the organisation has reflected Cochrane’s vision: Has it embraced the need to take an efficiency perspective?

'Allocation of funds and facilities are nearly always based on the opinion of consultants but, more and more, requests for additional facilities will have to be based on detailed arguments with 'hard evidence’ as to the gain to be expected from the patient’s angle **and the cost.** Few could possibly object to this’.

'If we are ever going to get the 'optimum’ results from our national expenditure on the NHS we must finally be able to express the results in the form of the benefit **and the cost** to the population of a particular type of activity, and the increased benefit that would be obtained if more money were made available’ [1].

Decisions based on highly focused evidence-based methodologies that consider only one dimension of relevant evidence (that is, whether the intervention works) may contribute to inefficient, or even wasteful, policy and practice. Equally, a decision based on an economic evaluation that does not utilise the most reliable evidence for effectiveness will also be flawed, just as an unsystematic review may lead to biased conclusions. A better approach is to explicitly consider the trade-offs between outcomes and cost. Studies of cost-effectiveness may arrive at different conclusions than studies that evaluate effectiveness and costs separately [2]; ideally, consideration is needed of both effectiveness and cost together to inform judgements on cost-effectiveness.

In this paper, we begin by briefly introducing economic evaluation. We review the current prevalence and quality of economic components of published Cochrane intervention reviews and summarise approaches to incorporating economic perspectives and evidence into such reviews. We trace key methodological developments during the first 20 years of The Cochrane Collaboration and highlight unresolved methodological issues that require further research.

### What is economic evaluation?

Economic evaluation involves the comparative analysis of alternative actions in terms of their costs and effects [3]. All types of economic evaluation seek to measure the costs of providing interventions and their broader cost consequences in the same way. The type of economic evaluation will vary according to the unit of measurement of benefit employed: for example, number of symptom events observed (when combined with cost data, this becomes a cost-effectiveness analysis); a measure of quality and quantity of life (cost-utility analysis), or outcomes expressed in monetary terms (cost-benefit analysis). Economic evaluation is used in many policy areas, and governments and other agencies have published methodological guidelines to help standardise conduct [4-9].

**Figure 1:**
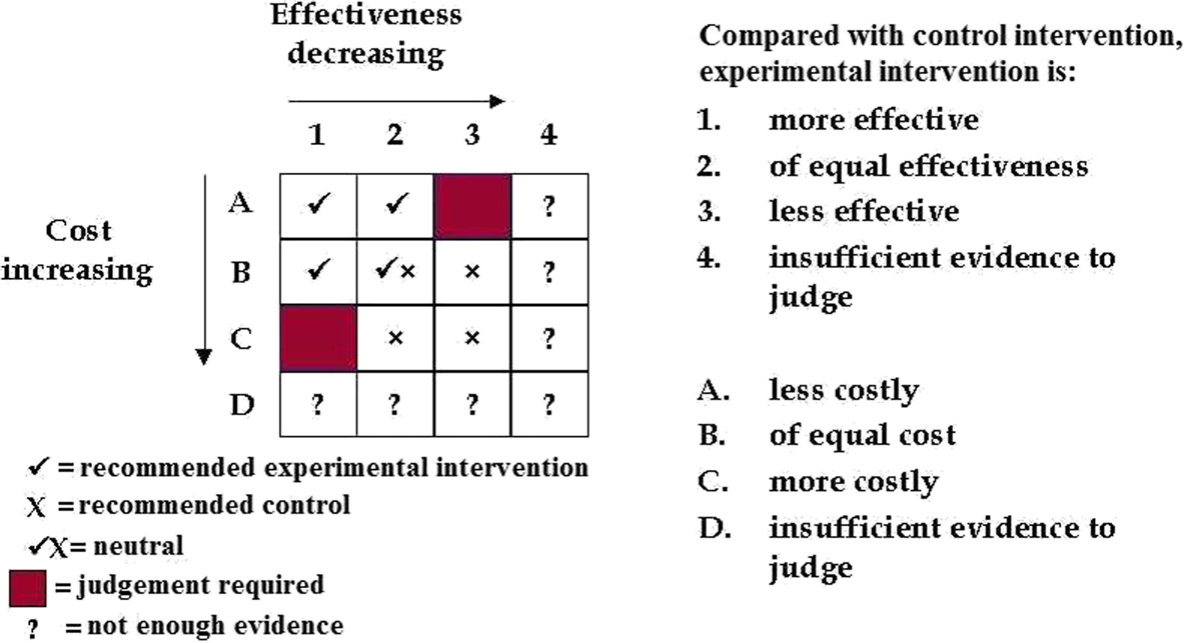
Decision-making relationship between effectiveness and cost*. *In this respect, we gratefully acknowledge other members of the Cochrane Health Economics Group: especially Ron Akehurst, Martin Buxton, Iain Chalmers, Ray Churnside, Paul Fenn, John Forbes, Alastair Gray, Jane Griffin, Sarah Howard, Tom Jefferson, Alastair McGuire, Bernie O’Brien, Andy Oxman and Adrian Towse who formulated this figure at the inaugural meeting of the group in 1993.

Figure 1 shows that, relative to current practice, a new health intervention could be (1) more effective, (2) of equal effectiveness or (3) less effective. Of course, a fourth option is possible whereby, after synthesising data collected from all relevant studies, there is insufficient evidence to conclude that the new intervention is more or less effective. Economists bring considerations of efficiency to the evaluation framework by adding the measurement of resources to that of effectiveness. We measure both the resources that are needed to provide the interventions under investigation (the resource inputs) and subsequent changes in the use of resources that occur as a consequence of using an intervention (resource consequences). For an economist the interest goes beyond the identification and measurement of these resources in natural units (for example, number of days in hospital) to consideration of the value of resources. Estimating the value of resources involves considering what benefits we could have obtained had we not given up the opportunity to use the same resources in another desirable way - this is the economic concept of opportunity cost. Returning to Figure 1, in terms of costs (that is, the monetised value of the resources used), a new intervention could be (A) less costly, (B) of equal cost, or (C) more costly, compared to current practice. (Again, there is the possibility of there being insufficient evidence to judge, as represented by row D).

For any intervention, the optimum position on the matrix is square A1, where the new intervention would both save costs and have greater effectiveness relative to current practice (and so is a 'recommended intervention’). A2 and B1 are also more efficient than current practice. B2 is neutral, with no difference in cost or effectiveness. In squares marked with an 'x’ the new procedure is less efficient, while those marked with a '?’ represent situations with insufficient evidence on effectiveness and/or costs to make a judgement. Of most interest are scenarios A3 and C1, where an important judgement must be made as to whether the additional cost associated with the more costly intervention is worth the additional effectiveness gained. To aid such a judgement an incremental value of the benefits gained can then be calculated along with an incremental value of the cost incurred to achieve such a gain.

### Cochrane reviews and economic evidence

In recent years, evidence has accumulated that policy-makers and other end-users would value more coverage of economic perspectives in systematic reviews, pointing to the sparseness of such evidence in Cochrane intervention reviews and related products as a major gap [3,10,11]. However, evidence on the quality and usefulness of economics components of published Cochrane intervention reviews remains limited. In 2006 and 2007, the Campbell and Cochrane Economics Methods Group (CCEMG) conducted an audit of economic components of all published Cochrane reviews [12]. This identified a range of approaches to incorporating economic perspectives and evidence that varied according to what costs and benefits were deemed relevant, along with inconsistencies between reviews in the application of economics methods at each stage of the review process. There were some examples of good practice, but also many examples of injudicious application of methods and interpretation of results. These findings informed the development of new methods guidelines for Cochrane contributors on whether and how to incorporate economics methods into the Cochrane review process at different levels [13]. As well as publication of methods guidelines in the Cochrane Handbook, a suite of companion training materials and tools for authors and editors have been made available at methods training workshops at annual Cochrane (and Campbell) colloquia and online via the CCEMG website (http://www.c-cemg.org).

One key tension is that as economic evaluations are conducted to inform specific decisions, some inputs to economic evaluations, including estimates of resource use and especially unit costs (that is, the opportunity cost of single units of resource use), vary between settings and over time [14]. Thus, the results of economic evaluations can have limited generalisability and transferability between settings or over time. Conversely (and while generalisability is also of equal concern for effectiveness components of reviews), the findings of Cochrane intervention reviews - including their economic components - are intended to be useful to a global audience of end users making specific decisions in different contexts.

This has led some commentators to question the value of incorporating economic perspectives and evidence into Cochrane reviews. We have long argued that such a conclusion is only valid if the economics components of a Cochrane intervention review were intended to produce definitive, widely applicable quantified estimates of the differences in resource use, costs and cost-effectiveness associated with the interventions under investigation. However, we argue that the starting point for economics components of Cochrane intervention reviews needs to be different. This is why Cochrane economics methods are not currently oriented towards developing decision analytic model-based economic evaluations as a further layer of evidence synthesis within a Cochrane review (although we argue that both economics and effectiveness components of reviews should be useful to inform such modelling exercises). Rather, the overall aim is to help end-users understand key economic trade-offs between alternative interventions, by summarising evidence for resource use, costs and cost-effectiveness collected from published economic evaluations conducted in different settings and at different times, and placing this in the context of the best available evidence for intervention effects [15,16]. Depending on the choice of methods framework (see below), this summary will be more or less detailed and may (in the case of a more detailed summary) include a: critical appraisal of eligible published economic evaluations; investigation of factors likely to drive variations between settings and over time; and (in both cases) a provisional assessment of the extent to which an intervention is likely to be judged favourably from an economic perspective.

## Methods

### Frameworks for incorporating economic perspectives and evidence

**Figure 2:**
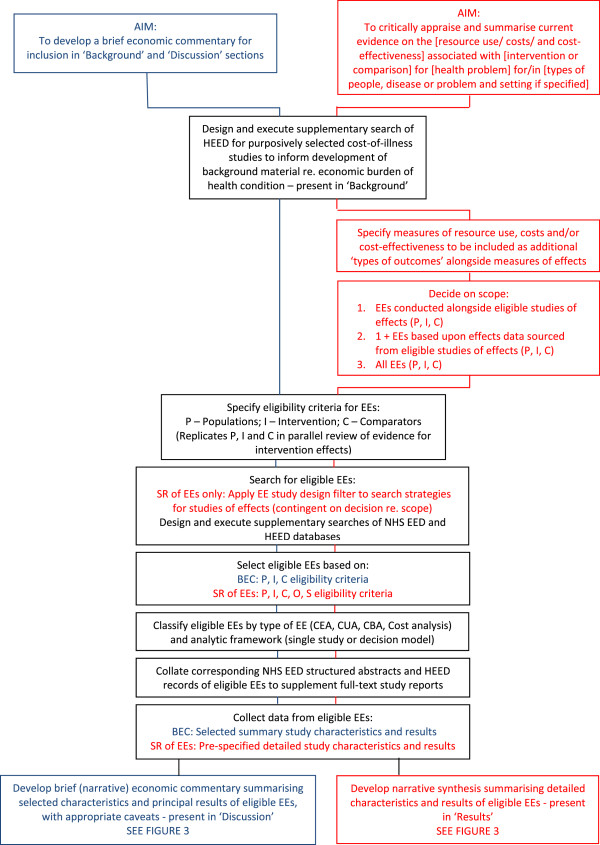
Framework for incorporating economic perspectives into Cochrane intervention reviews: aims and assembly of data.

**Figure 3:**
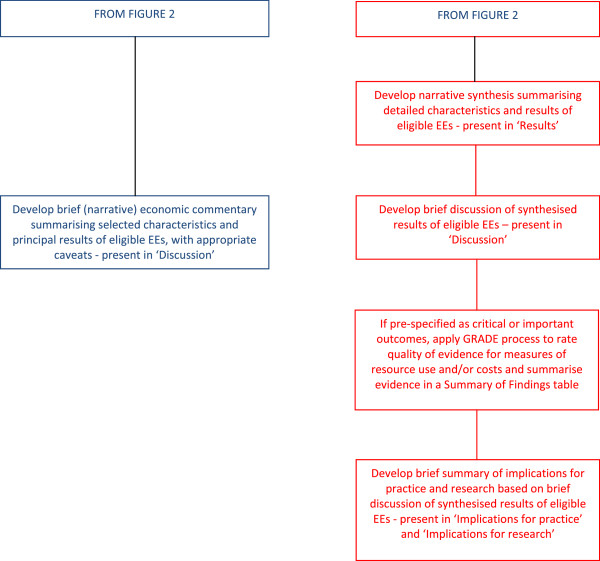
Framework for incorporating economic perspectives into Cochrane intervention reviews: presentation of economic data.

Two guiding frameworks for inclusion of economic perspectives in Cochrane reviews are currently offered. The first involves a full systematic review of evidence from previously published economic evaluations, integrated into the systematic review of evidence from studies of intervention effects. The second involves developing a brief economic commentary to be incorporated into the background and discussion sections of a Cochrane intervention review. These two frameworks share some common stages of the review process, as set out in Figures 2 and 3.

The two frameworks differ in terms of time and expertise needed to complete the respective processes. A full systematic review of economic evidence comprises precisely the same stages as a systematic review of evidence for effects and may add considerably to the workload of author teams producing the review (although in many cases the amount of available economic data will be limited). Author teams considering use of this framework are advised where possible to recruit an experienced health economist author or adviser, familiar with Cochrane review methods, from the outset, to lead or advise development of the economics component of the review. Authors should contact their Cochrane Review Group to check on the availability of a health economist advisor and, if there is not a nominated health economist, contact the CCEMG (http://www.c-cemg.org). Economics methods for conducting each stage of the systematic review process are described in Chapter 15 of the Cochrane Handbook [13] with a revised version being prepared for publication in 2014 to reflect recent advances in the methods. Further supporting guidance and tools for authors can be found on http://www.c-cemg.org.

A systematic review of economic evidence conducted alongside a review of evidence of intervention effects is recommended on three grounds. First, some of those effects assessed as part of the effectiveness review will also have impacts on resource use and associated costs. For example, a new surgical intervention that reduces rates of complications and revision procedures compared with current standard surgery will also lead to reductions in the quantities of resources needed to manage complications and perform revision procedures. Second, a key part of the assessment of risk of bias and methodological quality in published economic evaluations involves assessing published effect sizes used within the economic evaluation because such data are a subset of the data that might be considered by a systematic review of effectiveness. Third, a summary of evidence for impacts on resource use, costs and cost-effectiveness is most useful to end users of reviews when presented along with evidence for the direction and magnitude of intervention effects (as illustrated by Figure 1). We anticipate that possible future developments of Cochrane products may lead to this recommendation being reviewed. For example, technological developments may make it possible to link data to Cochrane content; this could allow modular reviews to be conducted separately and then linked to form bespoke packages of evidence requested by end users of The Cochrane Library. For instance, modules could be clustered around a 'standard model’ Cochrane intervention review and configured to address linked sets of questions about an intervention using different types of evidence.

Methods for developing brief economic commentaries have evolved recently, as a means of promoting the enhancement of Cochrane intervention reviews with more limited coverage of economic perspectives and evidence, without placing major additional burden (in term of expertise and workload) on Cochrane author teams and editorial bases. The process entails conducting supplementary searches of the NHS Economic Evaluation Database (NHS EED) and the Health Economic Evaluations Database (HEED) to identify full-text reports of relevant economic studies and their corresponding NHS EED and HEED records, and using the information they contain to develop brief, structured commentaries (full details of the approach can be found on http://www.c-cemg.org). Such an approach may also identify economic modelling studies that might be overlooked if the authors of such reviews limit their scope to economic evaluations conducted within the framework of single studies that meet eligibility criteria for the effectiveness component of the review (for example, economic evaluations conducted alongside included randomised controlled trials). Full details of recommended methods, including worked examples, and related training materials are available on CCEMG website (http://www.c-cemg.org).

Incorporating economic perspectives and evidence into Cochrane intervention reviews using either one of the frameworks introduced in this section is currently optional for Cochrane reviewers. It is therefore important to highlight that authors can still configure their Cochrane intervention reviews to help inform both the production of new economic evaluations, and considerations of economic issues by end users, even if they decide against developing a formal economics component using the frameworks described above. At the most basic level, authors can record bibliographic details of reports of population, intervention, comparator, outcome (PICO)-relevant published economic evaluations that they encounter when screening search results and selecting studies of effects, and present these in an appendix to the published Cochrane review, possibly annotated with links to corresponding NHS EED or HEED structured abstract records (if available). This would provide a useful resource for health economists and other analysts working with health technology assessment agencies, clinical guideline developers and other organisations that are tasked with developing new economic evaluations to inform specific decisions. Additionally, some Cochrane intervention reviews could benefit from consultation with a health economist towards the end of the review process, to place an 'economic lens’ on the reviewed evidence for effects. This is because it is conceivable that, by considering the balance of beneficial and adverse effects along with consideration of the cost of providing an intervention and the impact on use of services (often estimated as a marker of effectiveness), a judgement may be possible as to whether or not an intervention is likely to be considered favourably from an economic perspective. Although great care must be taken not to over-interpret a limited evidence base, placing an 'economic lens’ on the reviewed evidence for effects can allow tentative inferences to be drawn. An example of this was a recent study investigating the use of oesophageal Doppler monitoring to assess cardiac output and haemodynamic status, both considered key to improving outcome in high risk surgery and critically ill patients. No economic evaluations were available but the available data were organised into a series of balance sheets outlining the pros and cons of introducing this technology. Consideration of this evidence suggested that the introduction of oesophageal Doppler monitoring compared with conventional monitoring was likely to improve outcomes and that the upfront costs of providing oesophageal Doppler monitoring would be very likely to be offset by reductions in length of stay and the costs of managing adverse effects [17] (and available free on line at http://www.journalslibrary.nihr.ac.uk/hta/volume-13/issue-7).

In addition to considerations of resource use, costs and cost-effectiveness, providing an economic perspective can help end users of Cochrane reviews consider the implications of adopting an intervention in different settings. One way it can do this is by prompting the consideration of what resources would be required to implement or scale up interventions; interventions with the same apparent level of average cost-effectiveness can have very different budgetary implications. A second way is by prompting consideration of how costs and cost-effectiveness might vary between different population sub-groups. Consideration of how effectiveness might vary between sub-groups is commonly addressed in Cochrane intervention reviews, but the addition of an economic lens might help focus on 'economic’ reasons why behaviour differs between sub-groups in addition to biological or clinical reasons [18]. An example, of this might be to consider how the uptake and efficiency of public health interventions varies between socioeconomic groups according to the type and magnitude of financial incentives provided.

Cochrane reviews are only part of the evidence base required for decision-making. The Cochrane review process provides an opportunity to assist in the decision-making process in other ways. A positive spill-over (that is, a consequence of a course of action that is in addition to the one intended) from the Cochrane review process is the opportunity that they provide to collate information to aid development of new economic analyses. This may be particularly important in situations where there is a lack of previous economic evaluations of interventions. An example of such an area is the evaluation of public health interventions. In this area high quality economic evaluations are rare and further economic evaluation modelling may be needed. The studies identified within a Cochrane review may help inform the development of the disease and care pathways that would form the basis of a subsequent economic evaluation model. The conceptualisation of these pathways is a necessary precursor to the production of a high quality model; it also provides a framework which decision-makers can begin to create analyses applicable to their own jurisdiction. Ideally, other evidence might be used to produce these conceptual models but focusing disease and care pathways from studies included in the Cochrane intervention review also provides a mechanism to consider the applicability of evidence on effectiveness (and cost) from those studies for a specific context.

Likewise, Cochrane intervention reviews can also provide a mechanism for identifying evidence that might be used in a subsequent modelling exercise to inform the question 'Is it worth it?’ These data might include evidence of effectiveness, costs and the strength of preferences that patients and the public put on different outcomes ('utilities’ in economic parlance). It is likely that sufficient data to inform an economic model will not be available from the studies included in a Cochrane review and that further dedicated research to identify relevant robust information will be required. However, the Cochrane intervention review provides an initial low cost resource to identify such data.

## Results and discussion

### Cochrane economics methods: 1993 to 2013 and after 2013

As we reflect on the first 20 years of development of economics methods for Cochrane reviews, it is apposite to acknowledge the insight of those early leaders within The Cochrane Collaboration who recognised a need for the organisation to consider both efficiency and effectiveness perspectives. We should also acknowledge the legacy of those health economists who responded to this challenge (named in the legend to Figure 1) by forming, in 1993, the informal discussion group that evolved into the Campbell and Cochrane Economics Methods Group. However, the development and application of economics methods in Cochrane reviews has progressed at a much slower rate in comparison to the rapid growth in production of Cochrane reviews more generally. Barriers to this progress include: the specificity of economics methods applicable for use in Cochrane intervention reviews due to their global audience; limited availability of resources and capacity to support Cochrane methods development; and limited capacity and expertise to support the application of economics methods in reviews. In this context, major challenges remain to building capacity (through the training of Cochrane authors, editors and methodologists), to establishing a wider economics methods network to support production of economics components of Cochrane reviews, and to securing funds to support further economics methods development.

A number of notable milestones have been passed during the first 20 years of the Cochrane Collaboration with respect to economics. The NHS EED and HEED databases have been established as key resources for the economics components of reviews (both currently free at the point of use to Cochrane contributors). The Methods Group was co-registered with The Campbell Collaboration in 2003, expanding its scope to cover the applied fields of crime and justice, education, social welfare and (latterly) international development alongside health. Methods guidelines were first published in the Cochrane Handbook in 2008 [13]. A free online tool to automate the adjustment of estimates of costs for currency and price year was published in 2010 (http://eppi.ioe.ac.uk/costconversion/default.aspx). A book describing state-of-the-art approaches to evidence synthesis that combine economics and systematic review methods is now in its second edition [19,20]. The CCEMG has developed a new methods framework for brief economic commentaries (http://www.c-cemg.org). Most recently, methods guidelines have been published on the use of the GRADE system to rate quality of evidence for resource use and costs, which will facilitate the incorporation of economic evidence into Summary of Findings tables. These tables are increasingly used in Cochrane to summarise principal findings and quality of evidence for important outcomes [21].

Looking ahead to the next 20 years, the volume of available economic evaluation will increase as economic evaluations increasingly become required as part of trials and other comparative studies by funding and regulatory bodies. This growth of the evidence base indicates the increasing need to consider economic evidence by decision-makers. Methods for incorporating economic perspectives and evidence into Cochrane intervention reviews need to continue to evolve so that they better meet decision-makers needs [22]. More Cochrane reviews that utilise the methods frameworks we have outlined here are therefore urgently needed. The rate of progress will hinge on levels of organisational leadership, commitment to and investment in the production of economic components of Cochrane reviews, alongside levels of capacity and resources in the CCEMG, author teams, editorial bases and other Cochrane entities. Inevitably, further, sustained economics methods research and development are also needed. One major issue yet to be addressed is the development of methods guidelines for incorporating economic perspectives and evidence into Cochrane screening and diagnostic test accuracy reviews.

We next discuss some challenges to be addressed in the further development of economics methods for Cochrane intervention reviews (and methods for systematic reviews of economic evaluations more generally).

In recent work, Anderson and Shemilt discuss the possibility of producing pooled estimates of costs and cost-effectiveness when conducting systematic reviews of economic evaluations [15]. Arguing for a more explanatory approach to such reviews, they propose that the real contribution of a systematic review of economic evidence may not be to produce a single authoritative result, but to help decision-makers understand the structure of the resource allocation problem addressed and the impact on the overall result of key determinants of costs and cost-effectiveness. The methods frameworks described above in this paper are consistent with this view. Anderson and Shemilt further argue that systematic reviews of economic evaluations are likely to be most useful in: (i) identifying the most relevant study (for the decision problem in hand) for a particular setting; (ii) understanding the key economic trade-offs and causal relationships in a decision problem or policy area; or (iii) justifying and informing decision model development.

A consideration of each of these three points in turn demonstrates that further investigation is needed to clarify how best to identify key studies with results that are applicable or transferable to particular jurisdictions and key economic trade-offs. With respect to key studies the value of meta-analytic techniques to explore the impact of factors likely to explain variation (that is, investigate heterogeneity) in estimates of resource use, costs and effects between studies remains under-explored for economic data. A multivariate meta-regression analysis, in principle, allows the effects of multiple explanatory factors to be investigated simultaneously. Brunetti and colleagues have recently published brief guidance notes to inform judgements about whether generating and presenting pooled estimates of resource use and costs is likely to be appropriate, with a view to investigating pre-specified factors that may drive between-study heterogeneity in such estimates [21]. They suggest that meta-analysis of estimates of specific items of resource use may be judged appropriate provided that the metric used to quantify such estimates is common between studies (or a common metric can be derived), and that meta-analysis of estimates of costs may be judged appropriate in a more limited set of circumstances, and even then, only after estimates derived from different studies have first been adjusted to a common currency and price year. However, we are not aware of any examples which have used standard meta-analytic techniques to pool, and investigate 'between-study’ heterogeneity in published estimates of resources use and costs within a systematic review framework. The feasibility and usefulness of this approach therefore warrants further study.

It is clear that many health technology assessment organisations, for example, NICE in England [7], now rely on decision-analytic models to help assess the effectiveness and cost-effectiveness of interventions. Cochrane intervention reviews remain important inputs to this process but are not sufficient. This is because individual reviews do not include all relevant comparators, and Cochrane reviews do not include the further level of evidence synthesis that is provided by a decision-analytic model. To ensure that Cochrane reviews remain relevant to decision-making, the CCEMG needs to grapple with this issue, which has at its heart consideration of the transferability of findings. Some elements of a decision model are more likely to be transferable than others. A decision model describes two related processes - the disease pathway and a prevention/care pathway. The disease pathway is determined by underlying biology and may be more transferable, whilst the prevention/care pathway may be less transferable; but this does not preclude the formulation of illustrative pathways that could inform the development of context specific models in particular settings. Such an approach might be further enhanced should the concept of modular reviews be adopted; illustrative models might be presented within a module to assist in the development of models applicable to particular end users. Furthermore, whilst cost and utility data might have limited transferability (and relevance to some decision makers [23]) the results of an illustrative model might be specified in natural units (for example, number of visits, length of stay) and resulting health states (probabilities of death, survival impaired, or with no problems).

Multi-level modelling is an alternative analytic technique that may hold some promise for investigation of factors likely to explain variation in estimates of costs, effects and cost-effectiveness within a systematic review framework. Economic evaluations typically provide multiple estimates of resource use, costs and effects in the form of both a 'base case’ analysis and often extensive sensitivity and sub-group analyses. Boehler [24], used such techniques to explore the relative importance of different predictive factors for the costs, effects and cost-effectiveness of statins both within and between studies and also between countries. The analytical approaches are complex and results may be biased if sensitivity and sub-group analyses are selectively reported in the index studies. Therefore, as with use of meta-regression techniques, further exploration of multi-level modelling approaches is needed.

## Conclusions

### Summary of key points for researchers

Methods for incorporating economic perspectives and evidence into Cochrane intervention reviews are now well established. The choice to conduct a full systematic review of health economic evaluations, fully integrated with the parallel Cochrane review of intervention effects, is not one to take lightly and this will require substantive input from a health economist. The incorporation of a brief economic commentary offers a simpler alternative likely to be better suited to author teams with limited resources and access to specialist expertise.

It is important to emphasise that the purpose of producing economics components of Cochrane intervention reviews is not to identify a single precise estimate of incremental cost-effectiveness that is widely applicable to the full range of decisions faced by end users. It is highly unlikely that such an estimate could be transferable and, though meta-analysis of cost data is technically possible, the resultant pooled estimate is unlikely to be applicable in any setting. Rather, the focus of narrative and statistical approaches to the synthesis of economic evidence is to identify key determinants of resource use, costs and/or cost-effectiveness and to draw out how these determinants may be distributed within and between settings. With careful presentation and interpretation, this will allow end users to determine the implications of findings of economics components of reviews in their own settings and to inform their particular context-specific decisions.

Over 40 years ago Archie Cochrane recognised the need to consider efficiency as well as effectiveness [1]. The methods to do this have been developed and should be adopted more widely. Major challenges still exist and the Cochrane Collaboration should aim to be at the forefront of attempts to overcome them, in order to help ensure that the work of the Cochrane Collaboration remains of relevance to end users who have to make decisions in the face of limited resources.

## Abbreviations

CCEMG: Campbell & Cochrane Economics Methods Group; HEED: Health economic evaluation database; NHS EED: National health service economic evaluation database; NICE: National institute for health and clinical excellence; PICO: Population, intervention, comparator, outcome.

## Competing interests

All authors belong to organisations that conduct economic analyses which are funded by a range of public and private organisations. No author has any financial competing interests. No author has any non-financial competing interests.

## Authors’ contributions

LV drafted the initial version of the manuscript and undertook preparation of the final version. IS and DMcD conducted substantial revisions on the text. IS DMcD, KM, CH, EB, JM, MD, MM, LV have made substantial contributions to the conception and design of the paper, have been involved in drafting it or have revised it critically for important intellectual content. All authors read and approved the final version to be published.

## Authors’ information

Campbell and Cochrane Economic Methods Group: Ian Shemilt, David McDaid, Kevin Marsh, Catherine Henderson, Jacqueline Mallander, Mike Drummond, Miranda Mugford and Luke Vale.
